# Risk factors of brain metastasis during the course of EGFR-TKIs therapy for patients with EGFR-mutated advanced lung adenocarcinoma

**DOI:** 10.18632/oncotarget.11918

**Published:** 2016-09-08

**Authors:** Xiaoyan Ma, Hui Zhu, Hongbo Guo, Anqin Han, Haiyong Wang, Wang Jing, Yan Zhang, Li Kong, Jinming Yu

**Affiliations:** ^1^ School of Medicine and Life Sciences, University of Jinan, Jinan, Shandong, China; ^2^ Department of Radiation Oncology, Shandong Cancer Hospital Affiliated to Shandong University, Jinan, Shandong, China; ^3^ Shandong Academy of Medical Sciences, Jinan, Shandong, China; ^4^ Department of Thoracic Surgery, Shandong Cancer Hospital Affiliated to Shandong University, Jinan, Shandong, China; ^5^ Department of Medical Oncology, Shandong Cancer Hospital Affiliated to Shandong University, Jinan, Shandong, China

**Keywords:** brain metastasis, prophylactic cranial irradiation, risk factors, epidermal growth factor receptor, advanced lung adenocarcinoma

## Abstract

Controversial value of prophylactic cranial irradiation (PCI) in NSCLC in terms of survival benefit prompted us to explore the possible risk factors for brain metastasis (BM) during the course of EGFR-TKIs therapy from EGFR-mutated advanced lung adenocarcinoma and identify the potential population most likely to benefit from PCI, because BM remains a therapeutically challenging issue. We retrospectively reviewed the records of 134 patients with EGFR-mutated advanced lung adenocarcinoma between 2008 and 2012. The cumulative incidence of BM was calculated by the Kaplan-Meier method, and Multivariate Cox regression analysis was used to assess the independent risk factors for BM. Thirty-four patients (34/134, 25.4%) developed BM during the course of EGFR-TKIs therapy. Moreover, the Multivariate analysis indicated that age ≤ 53 years (HR: 2.751, 95 % CI: 1.326-5.707; *p* = 0.007), serum carcinoembryonic antigen (CEA) ≥ 23 ng/mL (HR: 3.197, 95 % CI: 1.512-6.758; *p* = 0.002) and EGFR exon 21 point mutations (HR: 2.769, 95 % CI: 1.355-5.659; *p*= 0.005) were the independent high-risk factors for developing BM, which could offer important insights into the individualized treatment. Further studies are warranted to validate our findings.

## INTRODUCTION

Lung cancer, including non-small cell lung cancer (NSCLC) and small cell lung cancer (SCLC), is the leading cause of cancer death all over the world [1]. Advanced lung adenocarcinoma is the predominant type of NSCLC and BM is a common problem in NSCLC with a dismal prognosis. Approximately 10% of NSCLC patients present with BM at their initial diagnosis and 40-50% of NSCLC patients develop BM during the course of the disease [2]. BM, undoubtedly, exerts a devastating influence on survival and quality of life of these patients; the median overall survival (mOS) was reported to be merely 4-6 months after whole brain radiation therapy (WBRT) [3, 4]. Therefore, special attention should be paid to reduce the incidence of BM.

For patients with EGFR-mutated NSCLC, a large amount of clinical trials have demonstrated the overwhelming superiority of EGFR-TKIs as a first-line treatment over the routine chemotherapy according to response rate (RR) and progression-free survival (PFS) [5-10]. In addition, Sperduto et al. have revealed that EGFR mutations profoundly influenced the survival of patients with BM from lung adenocarcinoma, presenting EGFR-positive patients with the median survival time of 25.1months [11]. It has also been reported that EGFR-TKIs are effective in BM from EGFR-mutated NSCLC [12-14]. Indeed, EGFR-TKIs could pass through brain-blood barrier (BBB) and accumulate in brain metastatic lesions [15]. However, there remain some patients developing BM during the course of EGFR-TKIs therapy. Lee et al. [16] found that 26% developed central nervous system (CNS) failure and 13% experienced isolated CNS failure among 166 patients with a clinical benefit to EGFR-TKIs.

Recently, the survival benefit conferred by PCI in SCLC has been established. With regard to NSCLC, PCI can decrease the occurrence of BM, whereas uncertainties still remain regarding to the survival advantage [17]. To our knowledge, RTOG-0214 is a randomized controlled trial to explore the value of PCI in NSCLC on the basis of combined-modality therapy [17]. However, the study was closed early due to slow accrual and reached only a third of the targeted accrual. Moreover, it failed to improve OS and its outcome should be interpreted cautiously, which suggests that PCI should be targeted patients with the highest risk for BM and based on satisfactory locoregional and extracranial control. Therefore, we suppose that PCI could bring survival benefit for patients with a high risk of BM during the course of EGFR-TKIs therapy from EGFR-mutated advanced lung adenocarcinoma.

To date, previous results on risk factors of BM in NSCLC are not completely consistent, which included non-squamous cell carcinoma [18], younger age [18-20], high serum CEA level [21], no adjuvant chemotherapy [19, 22] and various disease stages [19, 22]. Notably, patients with EGFR-mutated pulmonary adenocarcinoma show a higher likelihood of BM than those with wild-type EGFR [23]. However, the risk factors for developing BM during the course of EGFR-TKIs therapy from EGFR-mutated advanced lung adenocarcinoma were rarely evaluated.

In the present study, we reviewed 134 patients with EGFR-mutated advanced lung adenocarcinoma, investigated the possible risk factors for developing BM during the course of EGFR-TKIs therapy and tried to identify the potential patients most likely to benefit from PCI.

## RESULTS

### Patient characteristics

For 134 patients included in this study, patient characteristics are shown in Table 1. The median age was 59 years (range: 35-81 years). Among 134 patients with EGFR-mutated advanced lung adenocarcinoma, 34 (25.4%) patients developed BM during the course of EGFR-TKIs treatment and 100 (74.6%) patients didn't.

**Table 1:** Clinical characteristics of patients with EGFR-TKIs therapy

Characteristics	NO.	%
**Age (years old)**		
>53	85	63.4
≤53	49	36.6
**Gender**		
Male	59	44.0
Female	75	56.0
**KPS score**		
≥80	117	87.3
<80	17	12.7
**Smoking status**		
Yes	31	23.1
No	103	76.9
**CEA (ng/mL)**		
>23	77	57.5
≥23	57	42.5
**EGFR-TKIs treatment**		
First-line	27	20.2
Second-line	72	53.7
Third-line or multi-line	35	26.1
**No. of extracranial metastasis**		
0	19	14.2
1	74	55.2
2 or more	41	30.6
**Type of EGFR mutations**		
Exon 19 deletion mutations	78	58.2
Exon 21 point mutations	56	41.8
**Type of EGFR-TKIs**		
Erlotinib	79	59.0
Gefitinib	55	41.0
**Brain metastasis**		
Yes	34	25.4
No	100	74.6

Abbreviations: KPS, karnofsky performance status; CEA, carcinoembryonic antigen; EGFR-TKIs, Epidermal growth factor receptor tyrosine kinase inhibitors

### Survival

**Figure 1:**
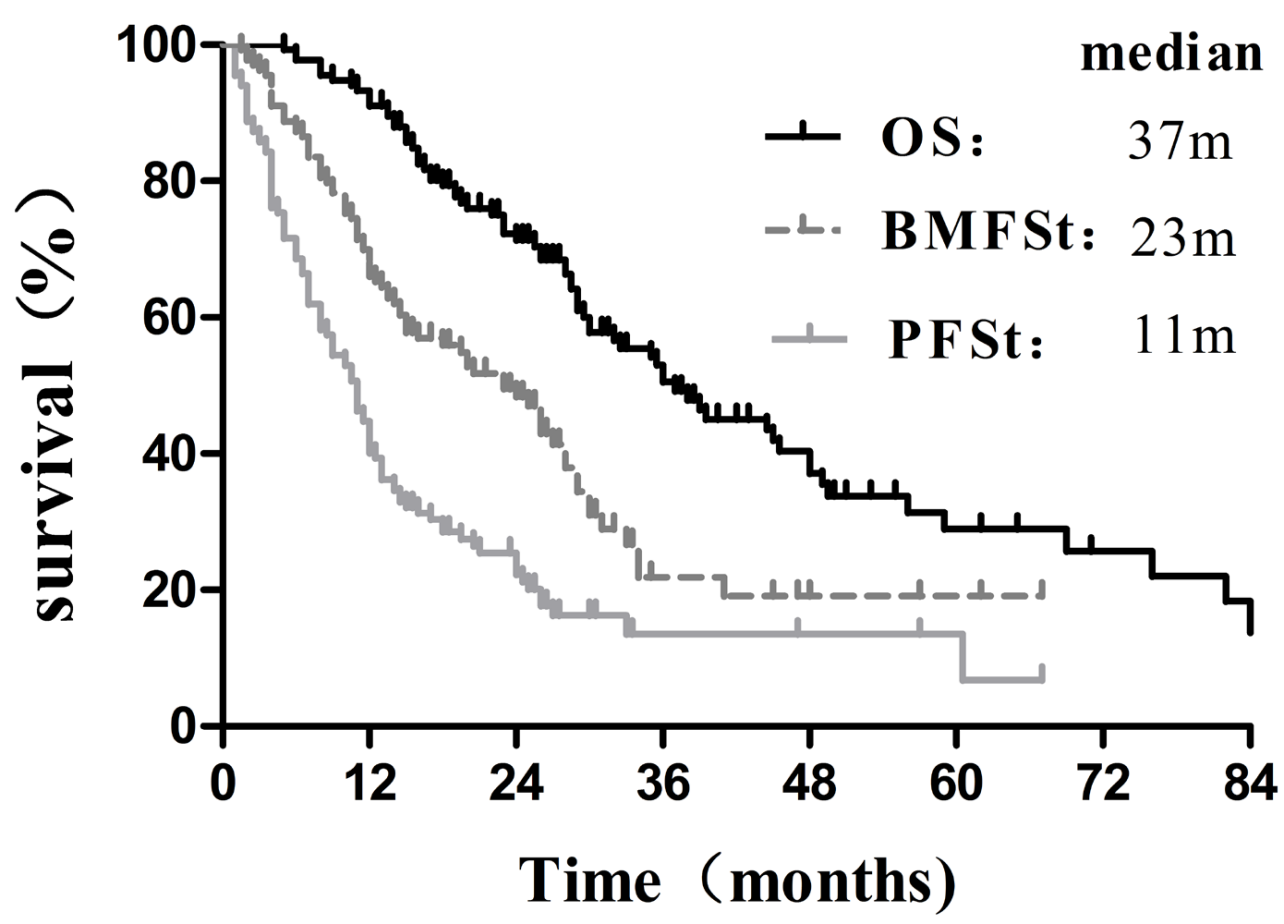
Overall survival, Brain-metastasis-free survival of TKIs and Progression-free survival of TKIs for 134 patients with EGFR-mutated advanced lung adenocarcinoma

**Figure 2:**
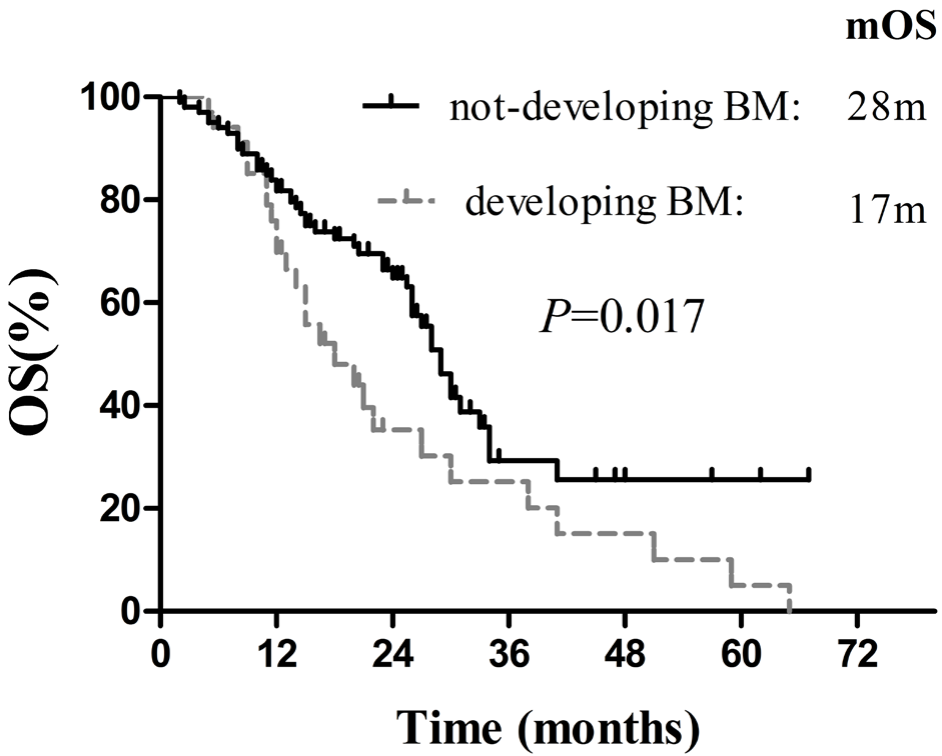
Comparison of the survival between patients with BM development and without BM development

Follow-up was completed in all 134 patients until December 2015, and the median follow-up period was 38.0months (range: 4.0-91.3 months). At the end of follow-up, 41 patients (30.6%) were still alive, whereas 93 patients (69.4%) had died. For these patients, median OS, median BMFS_t_ and median PFS_t_ were 37.0 months (95% CI: 27.1-46.9 months), 23.0 months (95% CI: 17.4-28.6 months) and 11.0 months (95% CI: 9.4-12.6 months), respectively (Figure 1). Additionally, the survival in these patients who didn't develop BM was significantly superior to that in patients who developed BM (*p* = 0.017, Figure 2), according to the calculation from the date of starting EGFR-TKIs treatment.

### BM and post-treatment

Thirty-four patients (34/134, 25.4%) developed BM during the course of EGFR-TKIs therapy. Among them, patients with symptomatic BM and asymptomatic BM were 28 (82.4%) and 6 (17.6%), respectively. In addition, 31 patients (31/34, 91.2%) receiving chemotherapy first developed BM during EGFR-TKIs therapy later. The median time for the occurrence of BM was 9 months (range: 1.5-25.0 months) on the basis of evaluation from the initiation of EGFR-TKIs treatment. The cumulative incidence of BM in all patients at 1 year and 2 years was 21.8% and 28.5%, respectively. The actuarial risk for the occurrence of BM was 38.8% (19/49) in patients with age ≤ 53 years and 17.6% (15/85) in patients with age > 53years; 33.9% (19/56) in patients harboring exon 21 point mutations and 19.2% (15/78) with exon 19 deletion mutations; and 38.6% (22/57) in patients with serum CEA ≥ 23 ng/mL and 15.6% (12/77) in the group with serum CEA < 23 ng/mL.

For 34 patients who developed BM during the course of EGFR-TKIs therapy, 26 patients (76.5%) experienced BM only, and 8 patients (23.5%) experienced BM and extracranial disease progression. Among them, 18 patients (52.9%) received radiation therapy (RT) plus continuous EGFR-TKIs, 8 patients (23.5%) switched to chemotherapy, 6 patients (17.6%) received continuous EGFR-TKIs and deferring RT until intracranial progression, and 2 patients (5.9%) received continuous EGFR-TKIs plus supportive care. With regard to 100 patients who didn't develop BM during the course of EGFR-TKIs therapy, excluding 21 cases with stable disease, the most common sites of extracranial disease progression were thoracic cavity (60.8%), bone (22.8%), liver (8.9%) and others (13.9%). Of them, 42 patients (53.2%) switched to chemotherapy, 27 patients (34.2%) received local therapy plus continuous EGFR-TKIs, and 10 patients (12.7%) received continuous EGFR-TKIs. As a result, the median duration of EGFR-TKIs therapy was 13.5 months (range: 1.0-67.0 months). Finally, the treatment responses were evaluated according to the RECIST 1.1 guidelines and categorized as complete response (CR), partial response (PR), stable disease (SD), and progressive disease (PD).

### Risk factors for BM development

**Figure 3:**
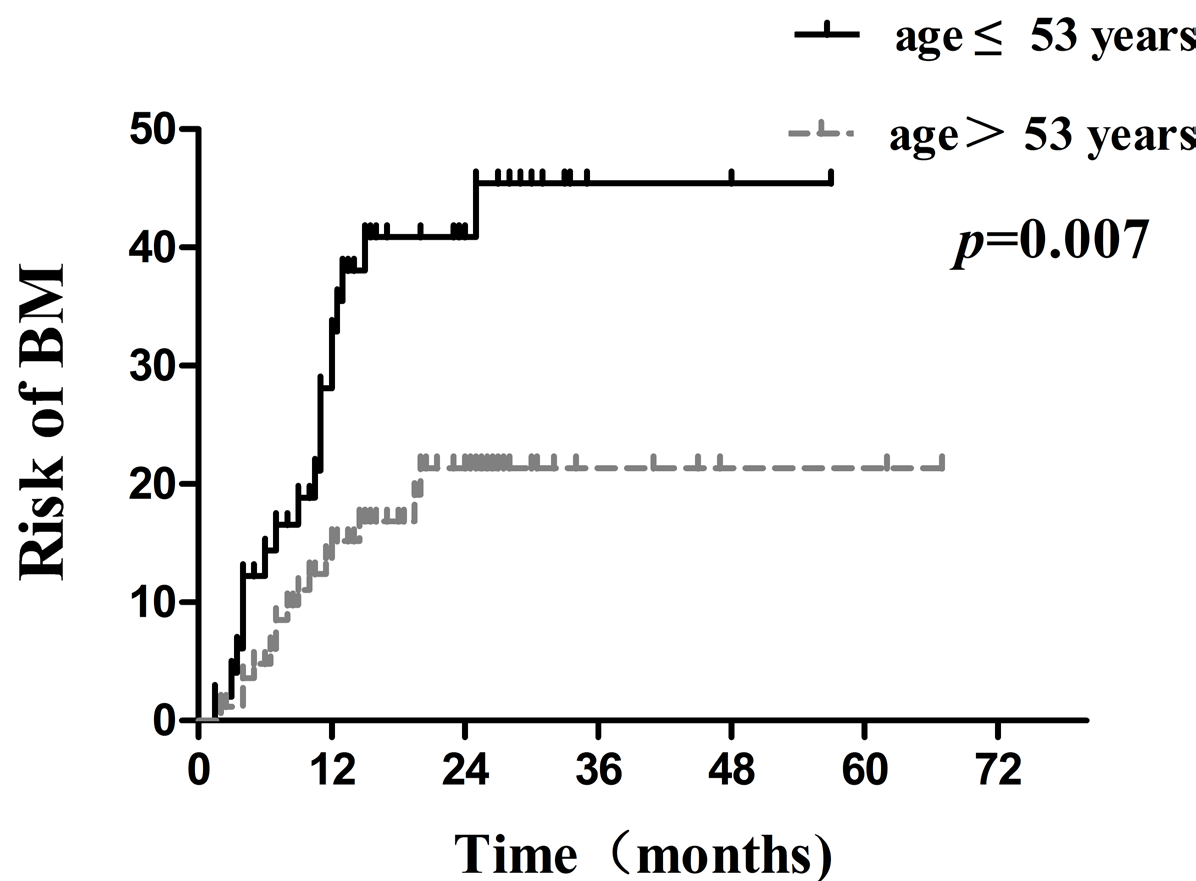
Comparison of the actuarial risk for developing BM between age > 53 years and age ≤ 53 years

**Figure 4:**
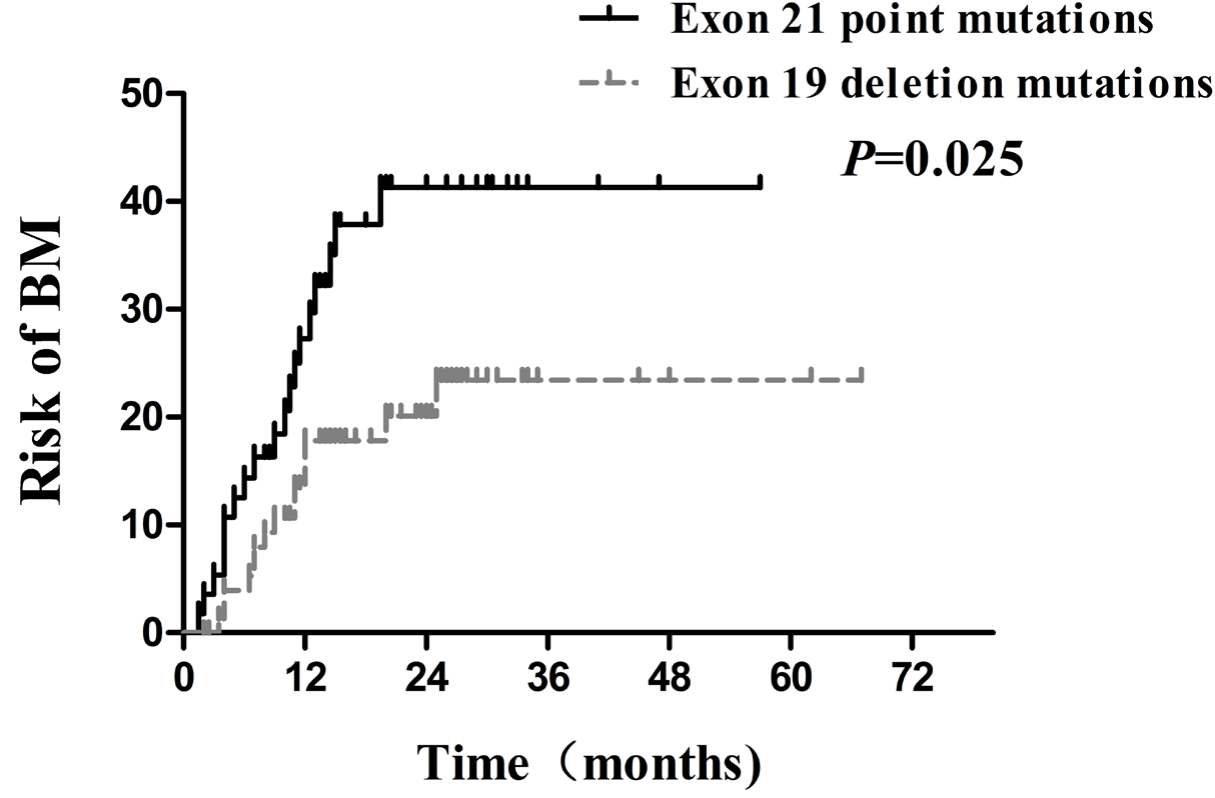
Comparison of the actuarial risk for developing BM between exon 21 point mutations and exon 19 deletion mutations

**Figure 5:**
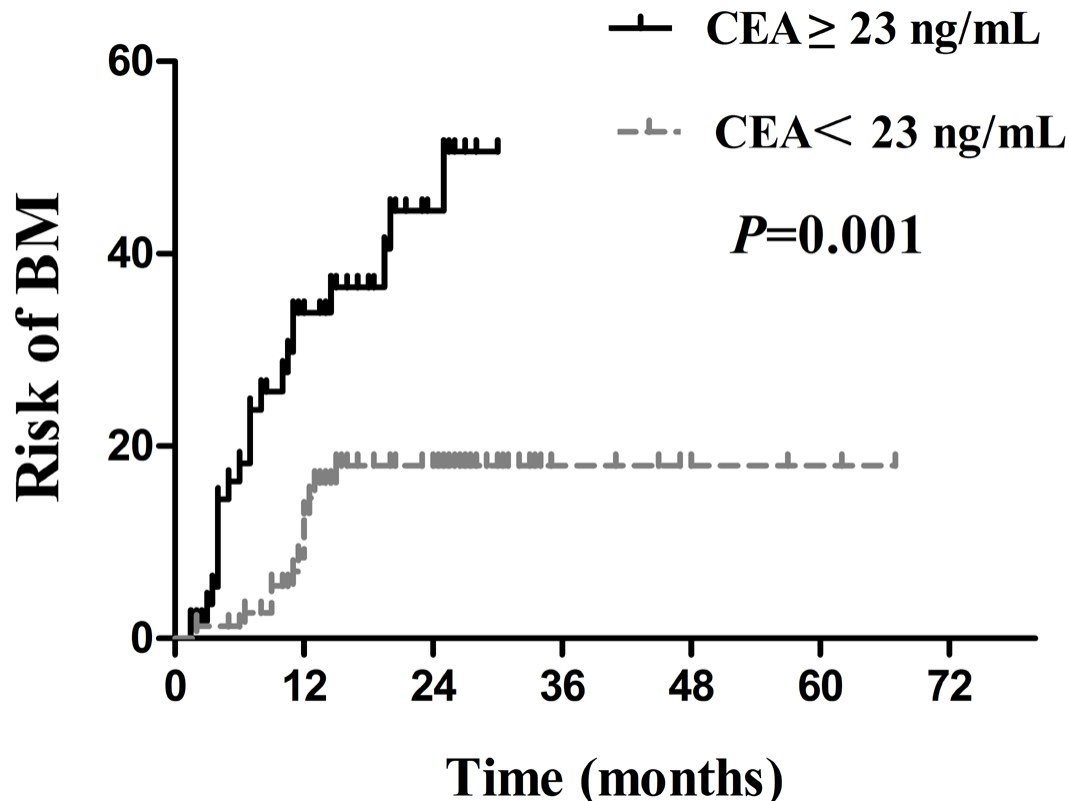
Comparison of the actuarial risk for developing BM between serum CEA ≥ 23 ng/mL and serum CEA < 23 ng/mL

**Figure 6:**
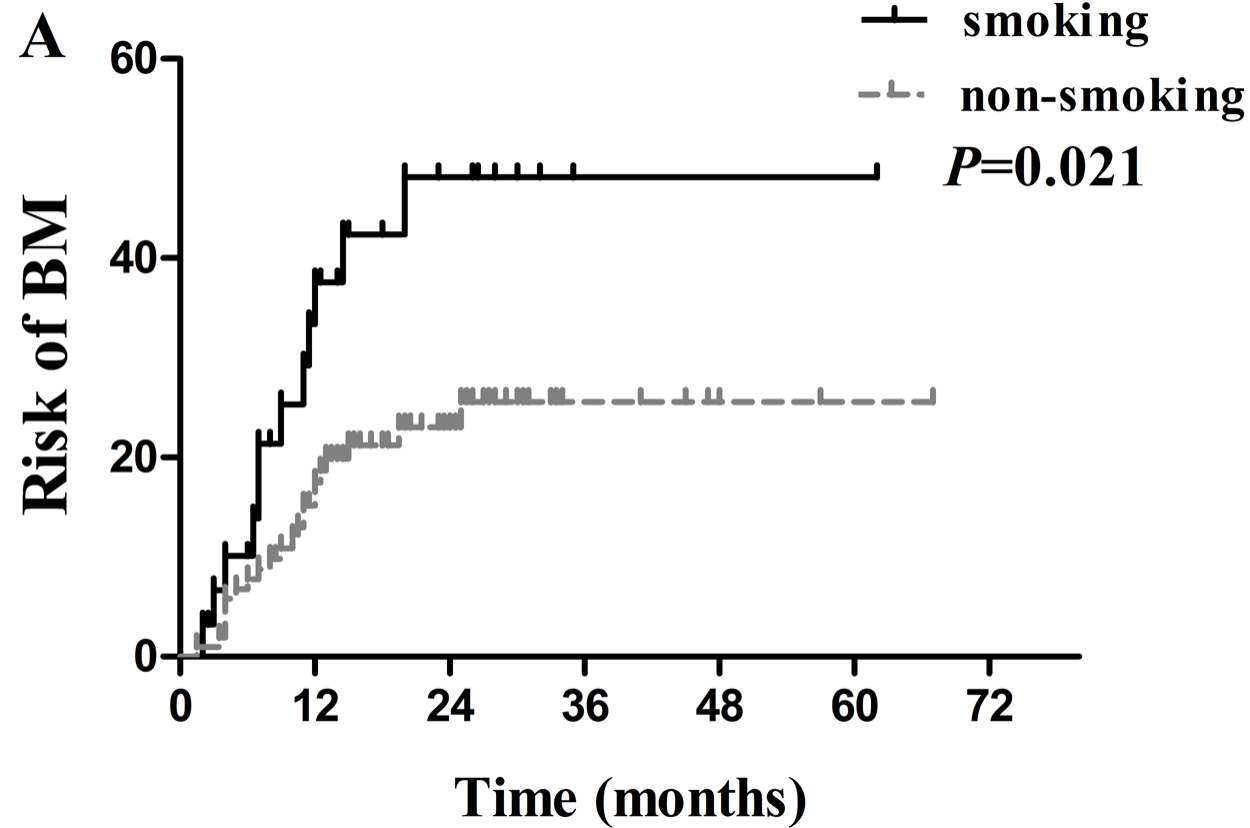
Comparison of the actuarial risk for developing BM **A.** between smoking and non-smoking; **B.** among patients with EGFR-TKIs first-line treatment, second-line treatment, and third-line or multi-line treatment.

Several clinical factors were observed to be associated with actuarial risk of developing BM by univariate and multivariate analyses in Table 2. In univariate analysis, the age of patients, type of EGFR mutations, serum CEA level, smoking status and treatment timing of EGFR-TKIs were associated with an increased risk of developing BM. The cumulative incidence of BM at 1 year and 2 years in patients with age ≤ 53years was 32.9% and 40.9%, respectively, which was significantly higher compared with15.2% and 21.3% respectively in patients with age > 53years ( *p* = 0.007; Figure 3). The 1- year and 2 -year actuarial risk of developing BM in patients with exon 21 point mutations was 28.1% and 41.3%, respectively, which was considerably higher compared with17.8% and 20.1% respectively in patients harboring exon 19 deletion mutations ( *p* = 0.025; Figure 4). The cumulative incidence of BM at 1 year and 2 years in patients with serum CEA ≥ 23 ng/mL was 33.9% and 44.5%, respectively, which was dramatically higher compared with 13.0% and 18.0% respectively in patients with serum CEA < 23 ng/mL ( *p* = 0.001; Figure 5). And the 1- year and 2 -year actuarial risk of developing BM in patients with smoking was 37.6% and 48.1% , respectively, which is higher compared with 17.5% and 23.0% respectively in patients with non-smoking ( *p* = 0.021; Figure 6A). Moreover, the actuarial risk of developing BM at 1 year and 2 years was 12.2% and 12.2%, respectively, in patients with first-line treatment; 21.1% and 24.5%, respectively, in patients with second-line treatment; and 31.2% and 49.8%, respectively, in patients with third-line or multi-line treatment ( *p* = 0.025, Figure 6B).

**Table 2:** Univariate and multivariate analyses for the factors associated with actuarial risks for BM

Factors	Univariate analysis Incidence of BM (%)				Multivariate analysis Incidence of BM (%)		
	1y	2y	X^2^	*P*	HR	95% CI	*P*
Age (years old)							
≤53	32.9	40.9					
>53	15.2	21.3	7.236	0.007	2.751	1.326-5.707	0.007
Gender							
Male	27.9	34.3					
Female	17.1	24.2	1.339	0.247			
KPS score							
≥80	19.2	25.7					
<80	39.6	47.1	2.957	0.086			
Smoking status							
Yes	37.6	48.1					
No	17.5	23.0	5.334	0.021	1.918	0.886-4.149	0.098
CEA (ng/mL)							
<23	13.0	18.0					
≥23	33.9	44.5	12.15	0.001	3.197	1.512-6.758	0.002
EGFR-TKIs treatment							
First-line	12.2	12.2					
Second-line	21.1	24.5					
Third-line or multi-line	31.2	49.8	7.343	0.025	1.276	0.722-2.257	0.401
No. of extracranial metastasis							
0	10.5	18.0					
1	22.8	26.0					
2 or more	25.8	41.3	1.758	0.415			
Type of EGFR mutations							
Exon 19 deletion mutations	17.8	20.1					
Exon 21 point mutations	28.1	41.3	5.055	0.025	2.769	1.355-5.659	0.005

Abbreviations: BM, brain metastasis

**Figure 7:**
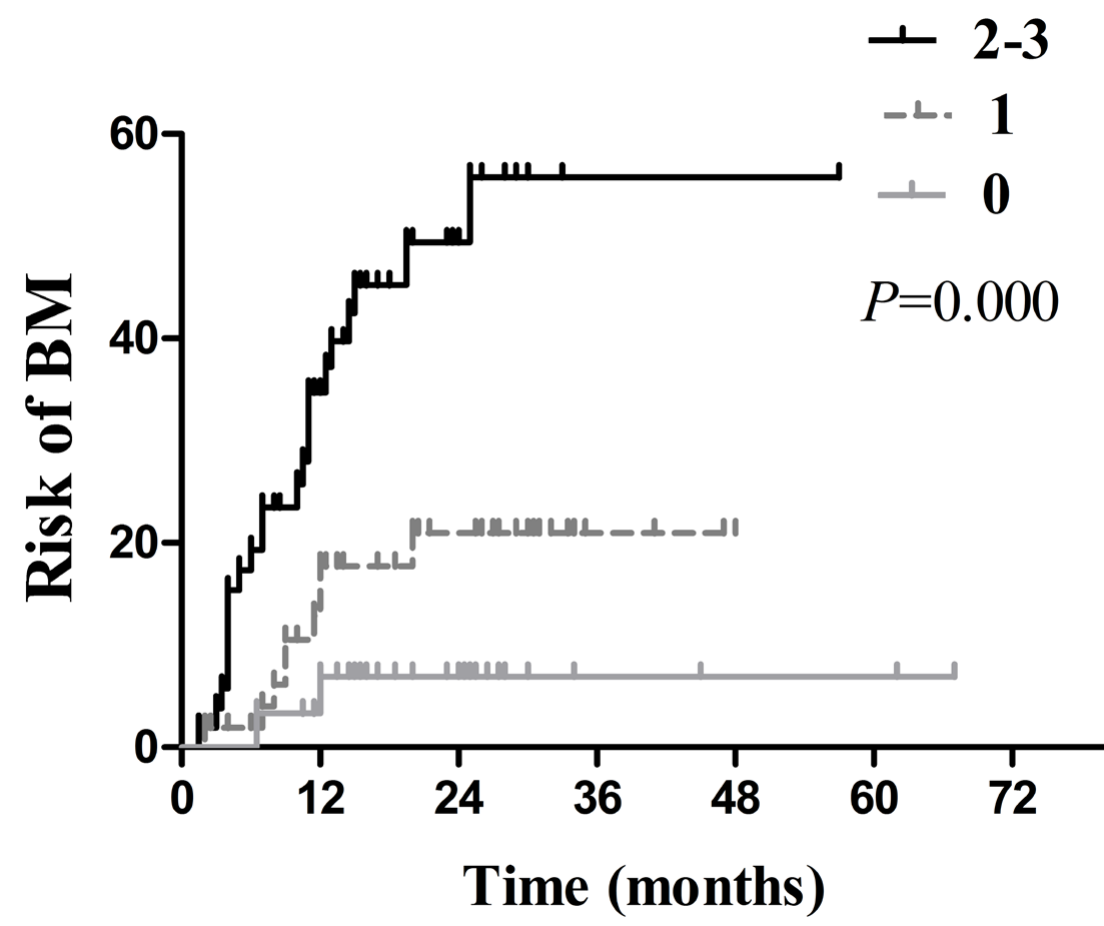
Comparison of the actuarial risk for developing BM among patients with different numbers of risk factors

Multivariate analysis indicated that age ≤ 53years (HR: 2.751, 95 % CI: 1.326-5.707; *p* = 0.007), exon 21 point mutations (HR: 2.769, 95 % CI: 1.355-5.659; *p* = 0.005), and serum CEA ≥ 23 ng/mL (HR: 3.197, 95 % CI: 1.512-6.758; *p* = 0.002) were independent high-risk factors of developing BM. In addition, further analysis and correlation on the independent risk factors above were conducted. For patients with no ( *n* = 30), 1 ( *n* = 52) and 2 or 3(*n* = 52) risk factors, the actuarial risk of developing BM at 1 year and 2 years were 6.9% and 6.9% , 17.7% and 21.0%, 34.7% and 49.4%, respectively ( *p* = 0.000, Figure 7). Obviously, patients with more risk factors are at a higher risk for developing BM.

## DISCUSSION

During the past decade, the advancement of EGFR-TKIs revolutionarily transformed the landscape of treatment and prognosis in advanced lung adenocarcinoma, but BM remains a therapeutically challenging issue [24]. BM from advanced lung adenocarcinoma, is a common reason leading to the failure of treatment, which often predicts poor prognosis [3, 4, 25]. On the other hand, there are still some patients developing BM during the course of EGFR-TKIs therapy, although EGFR-TKIs are effective for EGFR-mutated NSCLC patients with BM [12-14]. As reported in our study, thirty-four patients (34/134, 25.4%) developed BM during the course of EGFR-TKIs therapy. Moreover, patients had the tendency to live longer with more time to develop BM due to EGFR-TKIs therapy [26]. Therefore, it is urgent to identify the patients at the highest risk of BM for PCI, which may bring improvement in quality of life and survival for these patients.

Several studies have demonstrated that risk factors for BM in NSCLC [18-22], but there were no reports on the risk factors for developing BM during the course of EGFR-TKIs therapy from EGFR-mutated advanced lung adenocarcinoma. In the present study, multivariate analysis indicated that the age ≤ 53years was correlated with an increased risk of developing BM. Indeed, younger age was regarded as a high risk factor for BM in several studies. Bajard et al. [27] reported that age ≤ 62 years was a risk factor for BM (HR: 2.5, 95 % CI: 1.33-4.76; *p* = 0.004). Ceresoli et al. [28] assumed that age < 60 years was associated with a higher incidence rate of BM (HR: 1.26, 95 % CI: 1.03-1.53; *p* = 0.03). Similarly, Dimitropoulos et al. [29] confirmed that younger age (60.8 ± 8.9 years) was associated with a higher BM possibility (HR: 0.91; 95% CI:0.87-0.96; *p* < 0.001). In spite of the difference in the definition of younger age, to some extent, the results of these studies are consistent. So far, the reason that younger patients are at a higher risk for BM is still unclear. Younger patients with a higher risk for BM may be due to a more aggressive course of disease [30] and some biological factors, including high Ki-67 level, increased expression of vascular endothelial growth factor (VEGF) and so on [31, 32]. Further investigations are required to identify the specific reasons that younger patients are more likely to develop BM.

Recently, with the increasing availability of EGFR-TKIs, the type of EGFR mutations has become the research hotspot. Exon 19 deletion mutations and exon 21 point mutations, the most common EGFR mutations, account for approximately 85 % of all mutations of EGFR [33]. At present, several previous studies have shown that EGFR exon 19 or 21 mutations are different predictive markers for the response to EGFR-TKIs. It is well known that two open-label, randomized, phase III trials (LUX-Lung 3 and LUX-Lung 6) showed first-line afatinib improved OS compared with chemotherapy, especially in patients with exon 19 deletion mutation from lung adenocarcinoma but not in patients with exon 21 point mutations [10]. Sekine et al. [34] reported that the patients with exon 19 deletion mutations presented longer OS than those with exon 21 point mutations among NSCLC patients with erlotinib treatment after BM diagnosis ( *p* = 0.019). Furthermore, EGFR exon 19 or 21 mutations are now well recognized as different prognostic markers for NSCLC. A recent retrospective study by Li et al. [35] showed that exon 19 deletion, an independent prognostic factor, was associated with significantly longer survival in NSCLC with BM ( HR: 0.558, 95 % CI: 0.325-0.957; *p* = 0.034). According to our knowledge, previous studies usually focused on the predictive value of EGFR exon19 or 21 mutations in terms of the response to EGFR-TKIs and prognosis in NSCLC. In contrast, we investigated risk factors of BM development based on the type of EGFR mutations. In the present study, multivariate analysis demonstrated that exon 21 point mutations was an independent high-risk factor for BM (HR: 2.769, 95 % CI: 1.355-5.659; *p* = 0.005), which in turn supported previous studies. However, Heon et al. [36] retrospectively explored the risk of CNS progression in patients with IIIB/IV NSCLC initially treated with EGFR-TKIs, and drew an opposite conclusion that a higher risk of CNS progression was observed in patients bearing exon 19 deletion mutations. Accordingly, authoritative prospective investigations are required to validate the findings. The reasons for the significant difference between EGFR exon 19 deletion and 21 point mutations are still uncertain. One of the possible reasons for this finding may be attributed to different clinical characteristics and pathogenesis between exon 19 deletion and 21 point mutations, suggesting they were two kinds of NSCLCs [37]. Alternatively, exon 19 deletion mutations may lead to a better control of subclinical lesions of BM, which may be the explanation of lower incidence of BM.

CEA played an important role in the diagnosis, follow-up and prognosis of lung adenocarcinoma as an important tumor marker [38]. High serum CEA level was connected with tumor recurrence and metastases in resected NSCLC [39]. As the relationship between serum CEA levels and BM, Arrieta et al. [21] demonstrated that serum CEA ≥ 40 ng/mL is a risk factor for developing BM (HR:1.5, 95%CI:1.09-2.2; *p* = 0.014), particularly in lung adenocarcinoma. Consistent with the result above, our analysis for 134 patients with EGFR-mutated advanced lung adenocarcinoma also demonstrated that CEA ≥ 23 ng/mL was associated with an increased risk for developing BM. Taken together, high serum CEA level was not only related to tumor charge, but also suggested as a more invasive phenotype [21, 38]. Of note, the level of CEA in our study was measured prior to the initiation of EGFR-TKIs treatment, thus it can more accurately reflect the occurrence of BM during the course of EGFR-TKIs therapy.

There were no sufficient studies available regarding to treatment timing of EGFR-TKIs, especially in advanced NSCLC with BM. Koo et al. [39] reported that EGFR-TKIs are effective for EGFR-mutated lung adenocarcinoma, regardless of treatment timing. Among these 134 patients receiving EGFR-TKIs therapy in our study, 27 (20.2%), 72 (53.7%), and 35 (26.1%) patients received EGFR-TKIs as first-line, second-line, and third-line or multi-line treatment, respectively. The univariate analysis indicated that patients with third-line or multi-line treatment were more likely to develop BM (*p* = 0.025), whereas multivariate analysis failed to show a statistical difference in the association between treatment timing of EGFR-TKIs and BM development (*p* = 0.401). Whether there were some differences among first-line, second-line, and third-line or multi-line treatment in terms of BM development needs further exploration. Similar to treatment timing of EGFR-TKIs, only univariate analysis showed that smoking status was associated with a higher risk of developing BM. A study from Jain et al. [40] revealed that smoking status had negative impact on survival in a cohort of 211 patients with EGFR-mutated advanced lung adenocarcinoma treated with first line EGFR TKIs. Overall, published data are too limited to draw a definitive conclusion.

Currently, the profound significance of PCI in patients with limited SCLC and extensive SCLC has been established [41]. However, the survival advantage conferred by PCI in NSCLC remains controversial [17]. In this study, we struggled to identify the potential patients at the highest risk for BM during the course of EGFR-TKIs therapy for PCI, and the rationality of the investigation could be explained as follows. First, EGFR-TKIs as a systemic treatment have led to significant improvements in the control of locoregional and extracranial disease. Second, the combination of EGFR-TKIs and radiotherapy could result in favorable synergistic effects, including the radiosensitizing effect of TKIs and the opening of BBB by radiation [42]. Furthermore, the total dosage in the range of 20 to 40Gy may lead to the maximal opening of BBB, with the tolerable side effects [43]. Given all these considerations, PCI might bring survival benefit for targeted population.

In addition, median survival was 27 months (95% CI: 24.4-29.6 months) based on the calculation from the initiation of EGFR-TKIs treatment. The patients without BM development during the course of EGFR-TKIs therapy showed clear OS superiority over those with BM development. ( *p* = 0.017, Figure 2). Not surprisingly, for patients with more risk factors for BM, there was an obvious declining trend in OS ( *p* = 0.026). However, similar to other retrospective analyses, our study has several limitations. For example, EGFR mutation status was assessed using tissue specimens from primary and metastatic lesions, rather than BM. Importantly, the discordance rate of EGFR mutation status between primary tumors and corresponding metastases was reported to be 27- 28% [44], which may influence our findings to some extent. What's more, selection bias and heterogeneity in the enrollment may lead to a relatively longer OS, which was measured from the date of diagnosis. Undoubtedly, longer OS was mainly caused by 32 patients with postoperative recurrence or metastases.

## MATERIALS AND METHODS

### Patients

A total of 238 patients with EGFR-mutated advanced lung adenocarcinoma were treated with EGFR-TKIs at the Shandong Cancer Hospital and Institute between January 2008 and December 2012. The exclusion criteria were as follows: 1) BM identified before EGFR-TKIs therapy (90 patients); 2) EGFR-TKIs treatment less than one month (6 patients), and 3) incomplete clinical data to follow up (8 patients). Thus, 134 eligible patients were enrolled in the retrospective study, including 102 patients with stage IIIB-IV at initial diagnosis and32 patients with postoperative recurrence or metastases.These patients underwent a comprehensive assessment before EGFR-TKIs treatment, including physical examination, laboratory analysis, pathological type, EGFR mutation testing, and TNM stage. All patients had negative results of enhanced magnetic resonance imaging (MRI) or computed tomography (CT) scan of brain before EGFR-TKIs therapy; among these, 119 patients (88.8%) received brain enhanced MRI scan and 15 patients (11.2%) received brain enhanced CT scan. Generally, these patients periodically underwent a reexamination every two months, which composed of thoracic CT scan, abdomen B-ultrasound examination, brain enhanced MRI scan, and other necessary examinations based on their conditions. Of all the patients, 34 cases were identified to have BM during the course of EGFR-TKIs treatment. Additionally, EGFR mutations were detected by real-time fluorescent quantitative PCR (ARMS), using tissue specimens from primary and metastatic lesions. The study protocol was approved by the Ethics Committee of the Shandong Cancer Hospital and Institute.

### Treatment

Totally 134 patients with EGFR-mutated advanced lung adenocarcinoma received different treatment regimens before EGFR-TKIs therapy. Among these patients, 32 cases underwent postoperative recurrence or metastases and others were advanced NSCLC at the first present. There were 27 patients receiving EGFR-TKIs treatment as their first-line therapy, whereas the remaining preferred chemotherapy initially, with or without radiotherapy. For EGFR-TKIs therapy, gefitinib and erlotinib were continuously administered at the doses of 250 and 150 mg/day, respectively, until progression of disease (PD) or intolerable side effects.

### Statistical analysis

Brain-metastasis-free survival of TKIs (BMFS_t_) was defined as the time from the date of starting EGFR-TKIs treatment to the date of BM or to the date of last follow-up without the occurrence of BM. Progression-free survival of TKIs (PFS_t_) was defined as the time from the initiation of EGFR-TKIs therapy to disease progression or death. Overall survival (OS) was measured from the date of diagnosis to the date of death from any causes or the last known date that the patient was alive. The number of extracranial metastases was calculated by the region, such as thoracic cavity, bone, liver, adrenal glands and so on. In addition, the cut-off point of age and CEA were determined by ROC curve. The cumulative incidence of BM and survival were calculated by the Kaplan-Meier method. All statistically significant variables in univariate analysis were entered into the multivariate Cox regression analysis. The Log-rank test was used to compare the difference between groups, and two-sided *p* values < 0.05 was considered statistically significant.
